# Perceptions of environmental change and use of traditional knowledge to plan riparian forest restoration with relocated communities in Alcântara, Eastern Amazon

**DOI:** 10.1186/1746-4269-10-11

**Published:** 2014-01-27

**Authors:** Danielle Celentano, Guillaume Xavier Rousseau, Vera Lex Engel, Cristiane Lima Façanha, Elivaldo Moreira de Oliveira, Emanoel Gomes de Moura

**Affiliations:** 1 Amazonian Network for Biodiversity and Biotechnology Graduate Program (BIONORTE), Federal University of Maranhão (UFMA), Campus Universitário do Bacanga, Av. dos Portugueses, s/n, 65.000-000 São Luís, MA, Brazil; 2 Agroecology Graduate Program, Maranhão State University (UEMA), Campus Universitário Paulo VI, s/n, Tirirical, 65.054-970 São Luís, MA, Brazil; 3 São Paulo State University (UNESP), Campus of Botucatu, College of Agricultural Science (FCA), R. Dr. José Barbosa de Barros, 1780, 18.610-307 Botucatu, SP, Brazil; 4 Amazonian Network for Biodiversity and Biotechnology Graduate Program (BIONORTE), State University of Mato Grosso (UNEMAT), Av. Santos Dumont, s/n, Cidade Universitária - Celbe, DNER, 78.200-000 Cáceres, MT, Brazil; 5 Biodiversity and Conservation Graduate Program, Federal University of Maranhão (UFMA), Campus do Bacanga, Av. dos Portugueses, s/n, 65.000-000 São Luís, MA, Brazil

## Abstract

**Background:**

Riparian forests provide ecosystem services that are essential for human well-being. The Pepital River is the main water supply for Alcântara (Brazil) and its forests are disappearing. This is affecting water volume and distribution in the region. Promoting forest restoration is imperative. In deprived regions, restoration success depends on the integration of ecology, livelihoods and traditional knowledge (TEK). In this study, an interdisciplinary research framework is proposed to design riparian forest restoration strategies based on ecological data, TEK and social needs.

**Methods:**

This study takes place in a region presenting a complex history of human relocation and land tenure. Local populations from seven villages were surveyed to document livelihood (including ‘free-listing’ of agricultural crops and homegarden tree species). Additionally, their perceptions toward environmental changes were explored through semi-structured interviews (n = 79). Ethnobotanical information on forest species and their uses were assessed by local-specialists (n = 19). Remnants of conserved forests were surveyed to access ecological information on tree species (three plots of 1,000 m^2^). Results included descriptive statistics, frequency and Smith’s index of salience of the free-list results.

**Results:**

The local population depends primarily on slash-and-burn subsistence agriculture to meet their needs. Interviewees showed a strong empirical knowledge about the environmental problems of the river, and of their causes, consequences and potential solutions. Twenty-four tree species (dbh > 10 cm) were found at the reference sites. Tree density averaged 510 individuals per hectare (stdv = 91.6); and 12 species were considered the most abundant (density > 10ind/ha). There was a strong consensus among plant-specialists about the most important trees. The species lists from reference sites and plant-specialists presented an important convergence.

**Conclusions:**

Slash-and-burn agriculture is the main source of livelihood but also the main driver of forest degradation. Effective restoration approaches must transform problems into solutions by empowering local people. Successional agroforestry combining annual crops and trees may be a suitable transitional phase for restoration. The model must be designed collectively and include species of ecological, cultural, and socioeconomic value. In deprived communities of the Amazon, forest restoration must be a process that combines environmental and social gains.

## Abstract (pt)

**Resumo:**

As matas ciliares fornecem serviços ecossistêmicos essenciais ao bem-estar humano. O Rio Pepital é a principal fonte de água de Alcântara (Brasil) e suas matas estão desaparecendo, afetando o volume e a distribuição de água na região. Promover a restauração da mata ciliar do Rio Pepital é crucial. Em regiões carentes, o sucesso da restauração depende da integração da ecologia, dos meios de vida e dos conhecimentos tradicionais. Neste trabalho se propõe uma abordagem interdisciplinar para desenhar estratégias de restauração com base em dados ecológicos, conhecimento tradicional e as necessidades sociais. A área de estudo tem uma complexa história de realocação humana e de posse da terra. Populações de sete comunidades que usam ou usaram o Rio Pepital foram entrevistadas para documentar sua subsistência e percepções sobre as mudanças ambientais (n = 79). Informações etnobotânicas sobre espécies florestais nativas e seus usos foram avaliados com especialistas-locais (n = 19). Remanescentes de florestas conservadas foram avaliados para levantar informações ecológicas das espécies arbóreas (três parcelas de 1.000 m^2^). Os entrevistados mostraram um forte conhecimento empírico sobre os problemas ambientais do rio, suas causas, conseqüências e possíveis soluções. Foi encontrado um total de 24 espécies arbóreas (DAP > 10 cm) nas áreas de referência. A densidade de árvores média foi de 510 indivíduos por hectare (stdv = 91,6), sendo12 espécies consideradas as mais abundantes (densidade > 10ind/ha). Houve um alto consenso entre os especialistas locais sobre as espécies mais importantes da mata. As listas de espécies das áreas de referência e dos especialistas-locais apresentaram uma convergência importante. A agricultura de corte e queima é o principal meio de subsistência na bacia do Pepital e também a principal causa da degradação da mata ciliar. Para ter sucesso, a restauração deve transformar problemas em soluções, empoderando a população local. Dessa forma, os sistemas agroflorestais sucessionais combinando culturas anuais e árvores podem representar uma alternativa transitória para a restauração. O modelo deve ser construído coletivamente e incluir espécies de valores ecológicos, culturais e sócio-econômicos. Em comunidades carentes da Amazônia, a restauração florestal deve ser um processo que combina ganhos ambientais e sociais.

## Background

In the tropics, riparian forests are essential for human well-being. They provide ecosystem services such as water regulation, erosion control, forest products, fishery maintenance, biodiversity conservation, and leisure [1]. Despite being protected by law [2], these forests are widely threatened in Brazil by deforestation and competing anthropogenic activities, to such an extent that restoration efforts are urgently needed [3].

Ecological restoration is defined as “the process of assisting the recovery of an ecosystem that has been degraded, damaged or destroyed” [4]. It is an intentional activity that initiates or accelerates the recovery of an ecosystem with respect to its integrity, functionality and sustainability. According to Higgs [5], scientific and technological acumen is insufficient for restoration; it is necessary to respect other types of knowledge besides science, and especially to recognize the ethics and values that are beyond the scope of science. Participation of local communities in the planning and implementation of restoration efforts is essential [6,7]. Indeed, forest restoration success depends on the integration of ecology, livelihoods, and traditional knowledge [8]. Local people must be directly involved in the process of resource conservation, management and restoration.

Alcântara is a municipality in the Eastern Brazilian Amazon, on the northern Atlantic coast. In the early 80s, the Brazilian Space Agency started the construction of the Alcântara Launch Center (CLA) near the coast due to its advantageous geographical position for satellite launches [9]. For this reason, after two year of resistance, 312 families from 23 coastal communities were relocated from their traditional territories to inland *agrovillages* (rural villages planned and built by the government) [10]. The planning of human relocations did not fully consider social and environmental aspects. Communities that had fisheries as their main livelihood were placed far from the beach and were subjected to seashore access restrictions [11]. Moreover, many *agrovillages* were placed in the headwaters of the Pepital watershed.

The Pepital River is the main water supply for Alcântara. It is also very important for rural communities’ livelihood and recreation. Part of the relocated population used to live by the riverside close to the delta. The riparian forests of the Pepital River, especially in headwaters and main springs are highly degraded. The water level diminution in recent years has led to the commencement of water rationing in Alcântara. It is imperative to promote the restoration of the Pepital riparian forest. Part of the local community is aware of the problem and requested support from the Academia, which inspired the present proposal.

The rationale for this study is that an interdisciplinary research study based on ecological data, traditional ecological knowledge (TEK) and social needs is a suitable framework to design participative restoration efforts that combine ecological and social gains. To that end, relocated populations that use the Pepital River were surveyed to document their livelihoods, and to explore their perceptions toward environmental changes. Persons with remarkable knowledge of the local ecology were identified and consulted about plant species and forest restoration strategies. Finally, plots in conserved forests were surveyed to gain ecological information about species composition and abundance in non-degraded areas.

## Methods

### Study area

**Figure 1:**
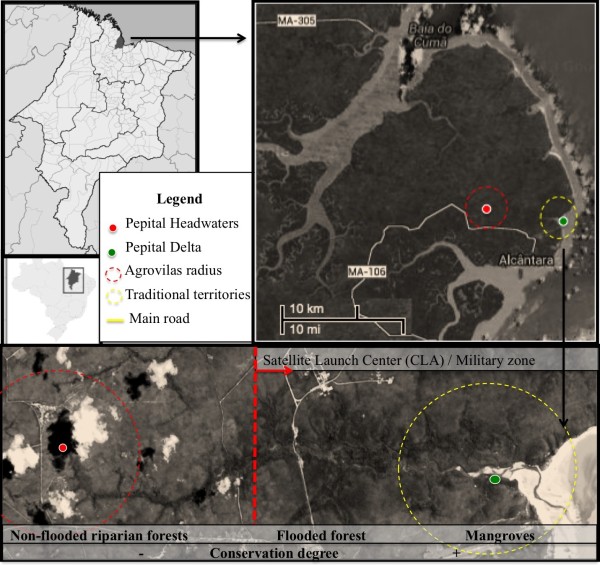
Maps and aerial photography (2013) showing the approximate location of the villages studied along the Pepital River, in Alcântara, Brazil (source: Google^®^).

The Pepital River watershed is located between latitude 2° 20′ and 2° 23′ S and longitude 44° 20′ and 44° 30′ W, in the municipality of Alcântara, Maranhão state, Brazil (Figure 1). This watershed is part of the Great Basin of the Atlantic and flows directly into the Atlantic Ocean. The study area is situated in the Amazon region. The soil type is low fertility Ultisol; the annual average precipitation is 2,000 mm with a distinct dry season from June to December and mean temperature of 25ºC. The original vegetation, typical of Eastern Amazon forests, features large trees and a well-defined understory [12]. It varies from non-flooded riparian forests (headwaters) to flooded forest and mangroves in the delta.

The study was conducted in seven villages (Agrovila Pepital, Maruda, Rio Grande, Cajueiro, Só Assim, Espera and Ponta Seca) that had received persons relocated from the Pepital River delta and vicinity in the early 80s (Figure 1). The distance of these villages to the Pepital headwaters varies from 0 to 7 km. Most relocated people used to live along the coast, close to the flooded forest and mangroves, where food resources from the forest, mangrove, river and beach were widely available [11]. They were displaced to areas close to the river headwaters and were given lands to cultivate (15 ha). According to Caldarelli [11], families from different communities were mixed upon relocation, thus breaking up longstanding neighborly and cultural relations. Access to their ancient lands was forbidden by the Alcantara Launch Center (CLA), which is controlled by the Brazilian Air Force because these areas are considered strategic for national security.

### Data collection and analyses

The research was undertaken between October 2012 and June 2013. A community meeting was held in the Agrovila Pepital to inform residents about the aims and methods of the study, and to ask for their participation in the research. Residents of the other villages surveyed were invited and transportation was made available.

Qualitative methods were used to assess information about livelihoods and local perception of river degradation, well-being and restoration needs. Semi-structured interviews were conducted with 79 households (23% of the village families). Interviews were always preceded by a presentation of the project and the team. The sampling was randomized. The first house of the village was visited and every fourth thereafter. When a house was empty it was replaced by the previous house; and if that one was also empty it was replaced with the next house in the village. Interviewed persons were preferentially old enough to remember the life and environmental conditions before the relocation process (age averaged 57 years old, with a standard deviation of 14.5). For this reason, eight questionnaires did not suit the criteria and were invalidated.

Interviews were conducted on a one-on-one basis and their duration varied between 40 minutes to 2 hours. Interviews consisted of asking relocated residents about four main subjects: A) their history, family, housing conditions, and perception of well-being; B) their livelihood and income (families that depended on agriculture and/or had agroforestry home gardens were asked to freely list the plant species they used); C) their present and past relation with the river (uses and benefits) and their perception about the situation of the river, its causes and consequences for human health; and D) their perception about forest restoration needs and potential approaches. These subjects were presented to the respondents through a standardized questionnaire that was filled out manually. The responses were carefully checked and grouped into categories defined afterwards for descriptive statistics such as percentages and frequencies.

Ethnobotanical information on native forest species and their use was assessed with 19 local specialists. These local specialists were identified among the respondents as the individuals that considered themselves and/or were considered by the community to be specialists in forest species [13]. With them the free-listing technique [14] was used by which they freely listed the tree species they knew to occur in the Pepital River riparian forest.

The free-list method is an efficient tool to indicate which species belong in the cultural domain [15]. Smith’s index is a measure of salience that ranges from 0 to 1 and is based on the highest frequency values and greater coincidence in the position of citation between informants, thereby promoting ordering of items in the list, which allows the identification of possible ‘breaks’ in the dataset [16]. A cultural consensus analysis of the data obtained from the free-list was performed to measure the degree of agreement among informants (“culturally correct” information) [17]. The frequency and Smith’s index of salience of the free-list results (agricultural crops, home garden trees species, wild fauna and tree species of the Pepital River) were calculated by ANTHROPAC 4.9 [15,18], and correlated at > 0.97. This high correlation between item frequency and Smith’s salience indicates consistency in the free-list results [19]. Only the species with Smith’s index higher than 0.1 were reported.

Field observations and ecological surveys provided complementary information. For the ecological survey, three plots of 1,000 m^2^ (50 × 20 m) were established in the most conserved riparian forests of the Pepital River to evaluate the relative richness and abundance of trees species (more than 10 individual per hectare). The location of the plots was set after a field trip carried out alongside the river led by two local specialists. Plots were at least 1 km apart from each other to guarantee sampling independence. In each plot, all trees >10 cm diameter at breast height (DBH) were sampled. The common names were given by the local specialists and samples were brought to the Maranhao State University (UEMA) herbarium to undergo botanical identification.

## Results and discussion

All informants belonged to the relocated community. The majority of them were female (58%); their age averaged 57 years (stdv = 14.5); and 63% had less than 3 years of formal education (most likely illiterate).

### Perception of well-being and livelihood in the *agrovillages*

In their traditional lands, families used to live in adobe houses without electricity, water supply or a sewer system. They were far from educational and health services, while public transportation was not available. In contrast, they had free access to abundant natural resources. In the *agrovillages*, relocated families received a brick house with electrical energy and water supply from a communal artesian well. They still do not have a sewer system or proper waste management (garbage is burned in 95% of homes). However, each *agrovillage* has a primary school and a medical clinic attended by a physician on a monthly basis. Roundtrip transportation is available daily to downtown Alcântara. Each family also received 15 ha for agriculture, but land tenure in these areas is insecure. Communities did not receive the property rights to these lands because the Alcantara Launch Center plans to expand in the future [11].

The majority of interviewees (65%) consider themselves to have a good quality of life, associating it with good infrastructure and security. For 26% of interviewed households, life quality is average. They claim that infrastructure, especially transportation is not sufficient (37%), or they complain that everything has to be purchased because of the distance to the river, forest and/or coast (63%). For 9% of the respondents, the quality of life in the *agrovillage* is poor and they are unanimous in the reason: everything has to be purchased due to the distance to the river, forest and/or coast.

According to historical documentation of the relocation process, *agrovillages*’ residents never reconciled with what had been done in their villages in relation to the management of natural resources [11]. There was an exchange route between villages that stopped after relocation because there was no longer surplus of production or fish; they became dependent upon money to purchase supplies [10]. There was a profound change in the surrounding environment, traditions and livelihoods of the relocated communities.

It is essential to identify the livelihood of a community before planning a restoration strategy, especially in regions where people suffer serious deprivation. Indeed, natural resource scarcity is often correlated with poverty and an unsustainable livelihood [20]. Most of the respondents depend on slash-and-burn agriculture (85.7%) to meet their livelihood needs. Only 15.7% of the householders depend directly upon the Alcantara Launch Center (CLA) and associated institutions for their familial income. Most of them formerly practiced slash-and-burn agriculture before relocation, but 17.1% fished as their main source of livelihood.

In the *agrovillages*, most of the people practice slash-and-burn subsistence agriculture (96.7%). Only 6.7% always have a surplus for sale in the community market while 31.7% sell sporadically. Interviewees mentioned twelve different crop species, but on average, each family cultivates four crop species (stdv = 1.4). Moreover, there is a strong consensus among interviewees about the seven preferred species (Smith Index > 0.1; Table 1). Cassava is the most preferred crop, probably because it is tolerant to low soil fertility and uncertain rainfall [21]. According to the FAO [22], cassava is the third most important source of calories in the tropics. It is grown mainly by poor farmers and often on marginal land. For local communities of the Pepital watershed, cassava is the main element of food security.

**Table 1:** Free list of agricultural plants species preferred by local population along the Pepital River in Alcântara, Brazil (n = 57)

**Portuguese common name**	**English name**	**Latin name**	**Frequency**	**Smith index**
Mandioca	Cassava	*Manihot esculenta*	40	0.671
Milho*	Maize	*Zea mais*	37	0.530
Arroz*	Rice	*Oryza sp.*	24	0.302
Melancia*	Watermellon	*Citrullus lanatus*	19	0.199
Quiabo*	Okra	*Abelmoschus esculentus*	18	0.182
Macaxeira	Manioc	*Manihot utilissima*	14	0.171
Maxixe*	Gherkin	*Cucumis anguria*	17	0.156
Pseudo-Reliability = 0.976				

*non-native species.

Homegardens play a key social-environmental role in the tropics, and are very important for smallholders’ food security and for providing ecosystem services [23]. At the study site, homegardens are important for 78.6% of the interviewees in providing fruits. During interviews, some householders complained of the absence of fruits in the garden or in proximity to the agrovillages. The trees were planted just after relocation and the gardens have started to increase fruit yield in the last 5 to 10 years. In total, they mentioned 36 different fruit species in their homegardens; but, on average, families have 4 fruit trees species in their homegarden (stdv = 1.3). Again, an important consensus was found in the eight preferred tree species (Smith Index > 0.1; Table 2). Mango is the most planted fruit tree. It was introduced to Brazil in the 16^th^ century by the Portuguese colonizers and is now widespread in the country [24]. In the Pepital watershed, mango is highly appreciated by the local population and it is very well adapted to soil and rainfall conditions.

**Table 2:** Free list of agroforestry homegarden plant species preferred by local population of the Pepital River in Alcântara, Brazil (n = 58)

**Portuguese name**	**English name**	**Latin name**	**Frequency**	**Smith index**
Mangueira*	Mango tree	*Mangifera sp*	31	0.497
Bananeira*	Banana tree	*Musa sp.*	30	0.335
Coqueiro*	Coconut tree	*Cocos nucifera*	15	0.213
Cajueiro	Cashew tree	*Anacardium occidentale*	13	0.199
Limoeiro*	Lemon tree	*Citrus sp.*	16	0.185
Cajazeiro	Caja tree	*Spondias mombin*	9	0.133
Laranjeira*	Orange tree	*Citrus sp.*	11	0.116
Tangerina*	Mandarin	*Citrus reticulata*	8	0.105
Pseudo-Reliability = 0.992				

*non-native species.

Even though most interviewees have an agricultural field and/or agroforestry homegarden, the majority of the respondents (88.6%) depend on complementary income from the federal government, such as a familial allowance for those who have children in school (45.7%), retirement pension (57.1%) or both (14.3%). This government subsidy represents the main source of net cash for the families.

### Perception of environmental change in the Pepital River

**Figure 2:**
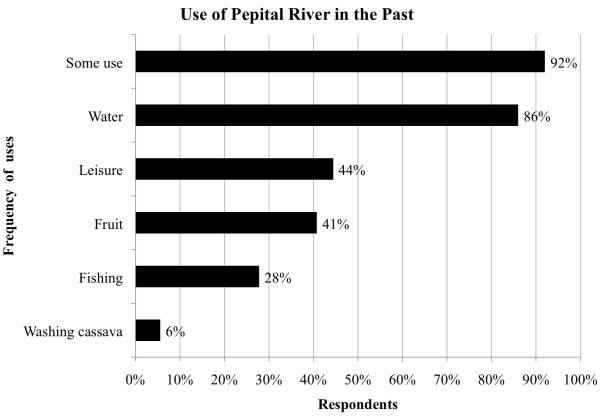
Pepital River uses before relocation process in Alcântara, Brazil (n = 59).

The Pepital River was an important element of community life before the relocation process according to the local people. Eighty-six percent of the respondents had known the river since their childhood and almost all interviewed households (92%) directly used the river before relocation (Figure 2) for water supply (86%), harvesting fruits (41%), leisure (44%), fishery (28%) or other motive. Today, 47% of those interviewed still use the river, especially for washing clothes when there is a problem with the community’s artesian pump. Nobody reported having continued to fish in the river, though some people still use it for leisure and some of the respondents walk toward the delta to collect fruits.

**Figure 3:**
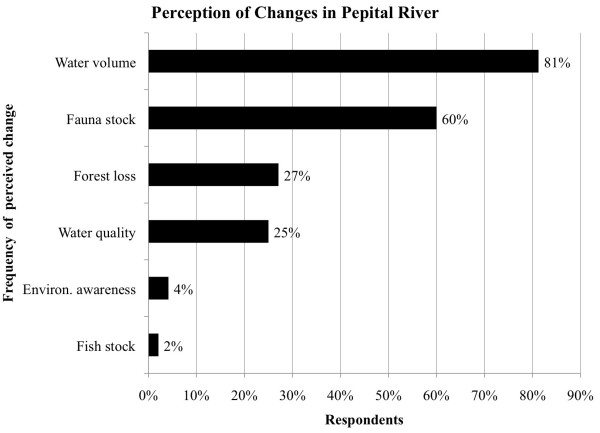
Perception of changes in the Pepital River in Alcântara, Brazil (n = 48).

More than half of the respondents (63%) consider the situation of the river poor or very poor. When asked what had changed in the river, 81% replied that water volume had decreased drastically since the relocation process (Figure 3). Other changes mentioned were: loss of forest (27%), deterioration of water quality (25%), decline in environmental awareness among the youth (4%), and decrease in fish stock (2%). Sixty percent of the interviewees mentioned an important decrease in native fauna stock. They listed 18 species that they used to find in the Pepital River riparian forest and expressed a strong consensus as to the nine most present in the collective memory (Smith index > 0.1; Table 3). All species were used for bush meat. All listed species (except caiman) play an important role in the equilibrium of the forest ecosystem as seed dispersers and may be used in the future for monitoring the success of ecological restoration efforts. It was observed that a large portion of the interviewed men are or were hunters, many of whom declared that they miss bush meat. Several informants mentioned that some wildlife species are now invading their fields to eat agricultural crops, which represents empirical evidence that the forest resources are declining.

**Table 3:** Free list of native fauna found in the past according to local population of the Pepital River in Alcântara, Brazil (n = 39)

**Portuguese common name**	**English name**	**Latin name**	**Frequency**	**Smith index**
Veado	Deer	Mazama sp.	20	0.353
Porco-do-Mato	Collared peccary	*Pecari tajacu*	17	0.335
Paca	Paca	*Cuniculus paca*	15	0.304
Tatu	Armadillo	*Tolypeutes tricinctus*	14	0.186
Macaco	Monkey	n.i. primates	8	0.154
Catitu	White-lipped peccary	*Tayassu pecari*	8	0.150
Jacaré	Caiman	n.i. Crocodylia	6	0.138
Cotia	Agouti	*Dasyproctidae prymnolopha*	9	0.130
Capivara	Capybara	*Hydrochoerus hydrochaeris*	6	0.102
Pseudo-Reliability = 0.976				

**Figure 4:**
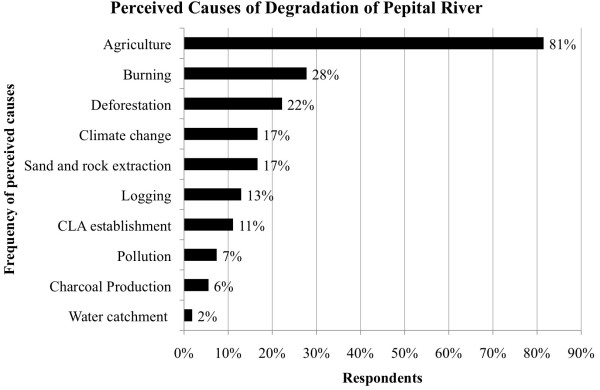
Perception of the causes of degradation of the Pepital River in Alcântara, Brazil (n = 54).

When asked about the causes of degradation, most of respondents (91%) mentioned at least one cause of destruction. For 11%, the Alcantara Launch Center (CLA) was the first one to deforest the Pepital riparian forest. They claim that the villages had been constructed by using wood and sand from the river. Agriculture was mentioned as a cause by 81% of respondents (Figure 4). Other quoted causes were: uncontrolled burning (28%), deforestation (22%), sand and rock extraction (17%), logging (13%), pollution (7%), and charcoal production (6%). For a minority (2%), water catchment for the city supply is one of the causes of the problem. Seventeen percent of those interviewed associated the river degradation with climate change (reduction of rainfall). In the year of interviews (2012), the local climate station reported less rainfall (970 mm) than the normal average (2,000 mm). Even though this outlier precipitation pattern probably affected the river volume that year, the decrease in the Pepital stream flow started to be perceived more than five years prior to the interviews (no secondary data on water volume was available). When asked about the impact of river degradation on human health, 86% of interviewees agreed that it affects human health because of water quality (69%) and/or associated diseases (14%).

### Degradation of Pepital River riparian forest

Forest degradation has different definitions [25]. This study considers riparian forest degradation the result of anthropogenic activity that decreases ecosystem productivity, biomass and biodiversity, and that is characterized by a reduction of tree density [25,26]. Deforestation and forest degradation in the Brazilian Amazon is a widespread problem [27] with a variety of negative impacts on environmental services [28] and human well-being [29]. The Pepital River riparian areas are a mosaic of lands in use for slash-and-burn agriculture, fallow with different ages of abandonment, and forests. The forest is constantly threatened by illegal activities (see next section) and the fallows (including species-rich young secondary forest) are constantly converted to slash-and-burn fields. According to Brazilian law, riparian forest must be preserved (or restored) and any other soil use is illegal [2].

Local people know that the Pepital River is disappearing and they have clear understanding that deforestation and forest degradation are the main causes. They also recognized that their livelihood (slash-and-burn subsistence agriculture) is the major driver of forest loss. Slash-and-burn agriculture (or shifting cultivation) is the dominant small-scale form of cultivation in the tropics [30]. In this system, farmers depend on the input of forest biomass ashes to fertilize their crops. Generally, the cycle is initiated by the logging of valuable timber while using other wood for charcoal production. Then, the impoverished forest area is burned and converted to an agricultural field. It is used for one or two production cycles and subsequently abandoned. After some years, the regenerated fallow is burned again. In Alcântara, as in many other areas of the tropics [30], the population increase leads to more pressure on the land and subsequent reduction of the fallow often below the natural soil fertility recovery time. The direct consequence is a reduction of yields and increased pressure on the land [30].

The slash-and-burn method provokes several negative environmental impacts, most notably air pollution and related respiratory diseases, uncontrolled fires, GES emissions, and loss of soil fertility when fallowing does not allow for proper soil restoration [30]. This method is especially harmful in riparian areas where it causes a severe depletion of ecosystem services such as water infiltration, erosion protection, biodiversity conservation, and carbon stock [1].

The use of riparian areas to cultivate under this regime is common because alluvial soils present the best fertility and moisture conditions [31,32], especially in the dry season between July and December. Even though Brazilian forest law forbids riparian forest conversion to agricultural uses, it is widespread on both small and large proprieties in the Amazon [33].

Moreover, the lack of security in land tenure of the relocated populations may also contribute to riparian forest deforestation. Land tenure issues are important factors driving environmental destruction in the Brazilian Amazon [34]. As mentioned before, each relocated family received 15 ha for cultivation but no title documenting property rights. Their utilization license can be repealed at any time in the future since Alcantara Launch Center plans to expand [11]. The “ownership” of Pepital River riparian areas is not clear. Some interviewees mentioned that such areas are the property of the Brazilian Air Force, whereas others stated that when the land was “given” by the government, they were asked to “respect” the riparian forest; however, 15 interviewees stated that they received land along the riverside to cultivate. Also, it was mentioned that the non-occupied riparian areas (areas not in agricultural use) are constantly “invaded” for extraction of resources or for establishment of new plots of slash-and-burn fields (facts evidenced in the field; see next section), which implies that some areas are treated as free-access without any external control. Some interviewees reported receiving areas inadequate for cultivation due to distance from their community (mean time to access their plots: 36.5 ± 31.2 minutes walking) and/or soil condition. Therefore, they occupied the riparian areas. In the words of an interviewee: “We know that cutting this forest is wrong, but when we need to feed our family we don’t think about it”. This statement justifies why they deforest and may help to guide restoration approaches that consider their livelihood needs.

### Non-degraded riparian forest: data from conserved areas and traditional ecological knowledge (TEK)

Determining reference conditions is a fundamental component for planning ecological restoration [4]. Overall, restoration planning begins with the selection of reference sites where comprehensive field sampling establishes attributes of the diversity and structure of native vegetation [35]. However, in disturbed landscapes TEK can make important contributions towards determining the composition and management of historical species [36].

**Figure 5:**
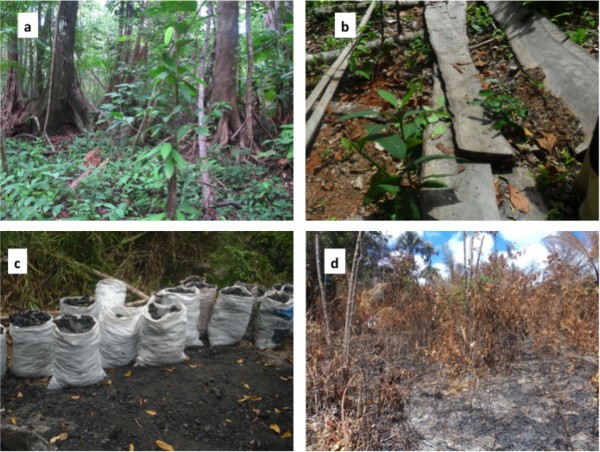
**Degradation of riparian forest in the Pepital River watershed in Alcântara, Brazil: a) Intact Forest (photography by D. Celentano), b) Logged tree (Alexandra da Piedade), c) Charcoal production (Ananda Asevedo; and d) Area recently burned for agriculture (Liliane Ribeiro).**

A field trip led by two local specialists along the entire Pepital River allowed three plots of 0.1 ha to be set up in the most conserved areas. This expedition revealed more areas of conserved forest areas than expected. Indeed, the most degraded areas are those close to the main headwaters (the first 6 km of the river) and community settlements as shown in a recent satellite image (see bottom of Figure 1). These areas are certainly the priority sites for restoration. The illegal practices mentioned by the interviewees (logging, recently burned areas, agricultural fields, and charcoal production) were evidenced in the field several times (Figure 5).

The most conserved forest areas (reference sites) constitute the only available parameter to elucidate what the non-degraded ecosystem was like and to guide future steps of restoration given the absence of surveys or botanical literature about the study sites based on information prior to degradation process (before the 1980s). In these areas were found a total of 24 tree species (dbh > 10 cm) from 16 families. Tree species richness averaged 12.7 (stdv of 3.2) while species density varied from 1 to almost 100 individuals per hectare. Total tree density was 510 individuals (dbh >10 cm) per hectare (stdv = 91.6). Trees averaged 21.7 ± 0.7 meters in height and 25.0 ± 4.8 cm in dbh. Twelve species were considered to be the most abundant tree species at the reference sites (density > 10 individuals per hectare, Table 4), and among those only one could not yet be identified (casca-grossa). The species accumulation curve did not stabilize with the three plots, indicating that additional samplings would increase the number of species. However, the most abundant and representative species of the conserved forest were captured by means of additional walking to find different species.

**Table 4:** Most abundant trees species (DBH > 10 cm) that occur in the most conserved riparian forests of the Pepital River at Alcântara, Brazil (n = 3 plots of 0.1 ha)

	**Botanical name**	**Family**	**Common name**	**Density (ind/hectare) mean ± stdv**	**Uses**
1	*Virola surinamensis* (Rol.) Warb.	Myristicaceae	Urucurana	97.3 ± 97.1	M, T, O
2	*Xylopia brasiliensis* Sprengel	Annonaceae	Pindaíba	63.3 ± 60.3	-
3	*Carapa guianesenis* Aubl.	Meliaceae	Andiroba	46.7 ± 50.3	M, T, O
4	*Symphonia globulifera* Linn. f.	Clusiaceae	Guanandi/Guananim	43.4 ± 40.4	M, T
5	*Mauritia flexuosa* L.F.	Arecaceae	Buriti	33.3 ± 32.1	F, S
6	*Euterpe oleraceae* Mart.	Arecaceae	Juçara/Açai	33.3 ± 43.6	F, H
7	*Richeria dressleri* G.L. Webster	Phyllanthaceae	Jaca-da-baixa	30.0 ± 43.6	-
8	*Anacardium sp.*	Anacardiaceae	Caju-da-baixa	26.7 ± 46.2	F
9	n.i.	n.i.	Casca-grossa	20.0 ± 34.6	-
10	*Attalea maripa* (Aubl.) Mart	Arecaceae	Inajá	16.7 ± 15.3	F, H, O
11	*Tapirira guianensis* Aubl.	Anacardiaceae	Pau-pombo	16.7 ± 15.3	-
12	*Annona sp*	Annonaceae	Araticum-da-baixa	13.3 ± 5.8	F

Legend of uses: *F *- fruit, *M*- medicinal, *O*– Oil, *T *– timber, *S*- straw, *H*– heart palm.

The interviewed local-specialists were the individuals that considered themselves and/or were considered by the community to be experts in the forest species. There were 19 key informants (13 males and 6 females) between the ages of 32 to 78 (average of 59.7 years and standard deviation of 10.7). They were asked to free-list the trees in the Pepital River riparian forest and afterwards to describe their uses. They mentioned 33 different trees species, but there was an important consensus (0.970) about the seven most important ones (Smith’s index > 0.1; Table 5). Among these species, only one (Mirim) does not coincide with the most abundant species at the reference sites (Table 4), where Mirim (*Humiria balsamifera*) was found at a low frequency (3.3 individuals per hectare, with an stdv of 5.8). Ten of the twelve species mentioned by the specialists but not found at the reference sites (data not presented) were mentioned only one time during free listing. When asked how they learned about the trees, they reported being taught from their parents, grandparents or elders from the community. When asked if their children also know, 72% replied yes.

**Table 5:** Free list of the main trees species of the Pepital riparian forest according to local plant specialists in Alcântara, Brazil (n = 19)

**Common name**	**Latin name**	**Family**	**Frequency**	**Smith index**	**Uses**
Guanandi	*Symphonia globulifera* Linn. f.	Clusiaceae	15	0.523	M, T
Urucurana	*Virola surinamensis* (Rol.) Warb.	Myristicaceae	14	0.506	M, T, O
Jussara/Açai	*Euterpe oleraceae* Mart.	Arecaceae	16	0.491	F, H
Buriti	*Mauritia flexuosa* L.F.	Arecaceae	15	0.404	F, S
Mirim	*Humiria balsamifera* (Aubl) St. Hill	Humiriaceae	7	0.273	
Pindaíba	*Xylopia brasiliensis* Sprengel	Annonaceae	6	0.187	
Andiroba	*Carapa guianesenis* Aubl.	Meliaceae	5	0.171	M, T, O
Pseudo-Reliability = 0.970					

Legend of uses: *F *- fruit, *M*- medicinal, *O*– oil, *T *– timber, *S*- straw, *H*– heart palm.

Most of the identified tree species have a direct human use as food or medicinal resource (Table 4). Moreover, four species (pindaíba, pau-pombo, urucurana and mirim) were mentioned as bearing fruits attractive to birds and other animals, which gives them a desirable characteristic for restoration projects. Mirim, urucurana, jussara and guanandi were mentioned several times as “water protectors”, i.e. trees that preserve water in the stream. Indeed, they were always located in flooded forest.

### Planning riparian forest restoration with the community

Restoration of degraded riparian areas is required by Brazilian law [2]. Restoration in human-modified landscapes must transform problems into solutions, involving and empowering local people. A proposed holistic concept of restoration was termed “Forest Landscape Restoration” (FLR), which is defined as the process that aims to restore ecological integrity and enhance human well-being in deforested or degraded forest landscapes [37]. This approach theoretically considers the dynamic and complex interactions between people, natural resources and land use on a landscape scale. It is crucial that it be considered in the Pepital River watershed, where slash-and-burn subsistence agriculture is the main livelihood (86% of families) and the major driver of riparian forest degradation. The local population is deprived and does not have many alternatives (or wishes) for drastically changing their way of life. However, they may be interested in designing a restoration program that considers their livelihood needs and restores their linkage with the river.

**Figure 6:**
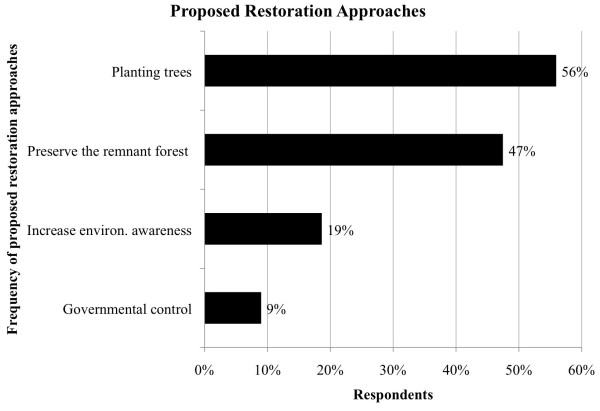
Proposed restoration approaches for the Pepital River in Alcântara, Brazil (n = 58).

When asked about forest restoration, more than half (58%) of the interviewees had never heard about it whereas the others had very little understanding. Just after this question, restoration was described through language easy to understand by them and respondents were almost unanimous on the need to restore the Pepital River riparian forest. When asked how it could be done, 74% of them replied (Figure 6) and the majority of respondents (56%) suggested planting trees. Furthermore, almost half of the interviewees (47%) claim that it is necessary to stop deforestation and preserve the remnant forest from slash-and-burn and other underlying causes of degradation. Indeed, restoration is a palliative solution where degradation of conserved forests still occurs [38]. Conserving the remaining standing forests is a priority in the Pepital watershed. First, there is a need to increase the environmental awareness within the local population and to augment the control over these areas by government, as 19% and 9%, respectively, of respondents suggested.

Planting trees is the main strategy to overcome tropical forest deforestation [39]. Interestingly, it is also common sense among more than half of interviewees (56%) as a strategy to restore the Pepital riparian forest. Restoration must consider ecological, social, and cultural aspects in its design. Traditional ecological knowledge (TEK) must be incorporated into restoration practices to account for cultural diversity and sustainable human participation in the ecosystem [40]. Moreover, people’s motivation and commitment to restoration projects is frequently related to species usefulness and desired environmental goods and services [41,42]. It is especially important in poor communities where livelihood drives environmental degradation as in the Pepital watershed.

Vieira et al. [8] proposed an agro-successional restoration approach, which they defined as incorporating a range of agroecology and agroforestry techniques as a transitional phase early in the forest restoration process. In this system, crops are planted at the same time as native tree species. Considering the livelihood needs of communities of the Pepital River and the local environmental conditions, the proposed approach may represent a viable way to incorporate species of ecological and cultural value as well as to consider livelihood needs, thus overcoming socioeconomic and ecological obstacles. A recently adopted Brazilian law now permits the use of agroforestry systems as a transitional restoration stage in riparian areas [43]. This approach permits landholders to use their livelihoods as a way to mitigate the damage and restore the landscape. As presented in the previous section, besides agriculture, homegardens are also important to householder food-security strategy (79%), as a means of providing fruits.

**Figure 7:**
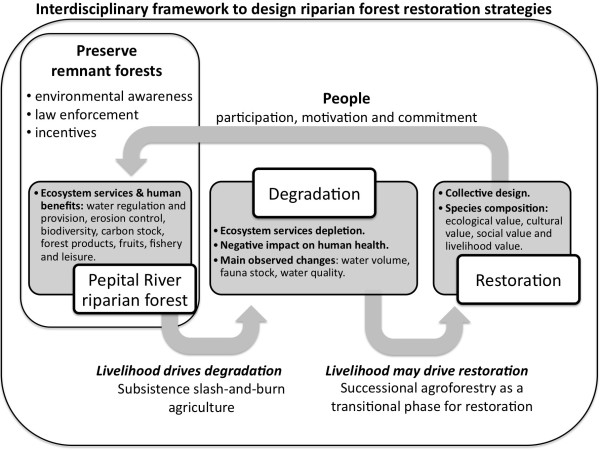
Flow chart showing the proposed interdisciplinary framework based on the present research results to design riparian forest restoration strategies along the Pepital River in Alcântara, Brazil.

Within this framework, successional agroforestry has also been proposed as a transitional phase for riparian forest restoration. The system (composition, arrangement, timing) must be designed collectively and discussions on species composition should be based on lists resulting from participatory research (Figure 7): i) trees that occur at conserved reference sites (ecological value); ii) species listed by local specialists (TEK, cultural value); iii) home-garden preferred trees (social value); iv) preferred annual crop plants providing food security (livelihood value).

Brazilian law forbids logging in riparian forests, even of planted trees. However, riparian forests can be managed for fruits, other non-timber forest products (NTFP) and annual crops (in the transitional phase). During interviews, local community listed the preferred crop species that interviewees grow in their fields (Table 1) and the tree species that they use in their agroforestry gardens, especially for fruits (Table 2). These lists will serve to illustrate and give foundation to participatory processes for choosing the species that are adapted to the specific conditions. During the initial restoration period, which might range from 3 to 20 years depending on the system [8], crops will be harvested, planted trees must be managed, and other forest species will regenerate naturally. As the canopy closes, agricultural crops will decline until they are removed. At this point, fruit trees will be producing and native forest species regenerating.

The use of non-native plant species in restoration has been criticized severely by some authors but acclaimed by others [44]. Considering the situation of the Pepital River, incorporating non-invasive useful exotic species into the restoration design might not be an ecological danger and may promote the engagement of the local community in the restoration process while diminishing the pressure upon conserved forests. The decision about which species to plant, as well as the specified planting design, may be built collectively in participatory workshops. However, it is strongly recommended that an interdisciplinary team – composed of agronomists, soil scientists, hydrologists, anthropologists, ecologists and other professionals – participate in the restoration efforts along with the local community. Potentially invasive species shall not be introduced in the system and the management of crop species must be based on agroecological and organic techniques, since chemical fertilizers, pesticides and other agrochemicals may contaminate the watercourse. The restoration plan may pre-define the elimination of exotic species at some point of the process.

The proposed restoration approach may be complemented by additional techniques, such as passive restoration [45] and enrichment plantings of less abundant and rare native species [46], for which a complementary botanical survey would be necessary. Moreover, critical areas (such as water sources and bluffs) may be restored for ecological purposes only (*sensu stricto* restoration). It is essential to monitor restoration throughout the process according to an adaptive management appraisal [47] by learning from the system outcomes and continually improving the practices to achieve ecological and social goals. Complementary actions (such as courses and workshops) to increase environmental awareness as well as to empower the local people in agroforestry, agroecology and restoration may be promoted. The presented restoration proposal, as any participatory approach, may or may not be accepted and/or adopted by the local community. If adopted, this system may increase the resilience of social-ecological systems. This approach may be suitable in other regions where poverty and livelihood drive environmental degradation. The research must be participatory and its results must always be validated and agreed upon by the community.

## Conclusions

In the present study an interdisciplinary research framework is proposed to design riparian forest restoration strategies based on ecological data, TEK and social needs. Firstly, the livelihood of the communities and preferred plant species (crops and agroforest trees) were documented; the past and present importance of the Pepital River for the communities and their perceptions of environmental change and causes for ecological damage were identified. Conserved forests were surveyed as reference sites and local specialists interviewed about the diversity of tree species.

Local populations showed a strong empirical knowledge of not only the environment problems of the Pepital River, but also of their causes, consequences and potential solutions. Although slash-and-burn agriculture is the main livelihood of this population, it is also the main driver of the Pepital River riparian forest degradation. An effective restoration approach must transform problems into solutions by involving and empowering local people. Successional agroforestry that mixes crops and trees may be a suitable transitional phase toward restoration. The model must be designed collectively and include species of ecological, cultural and socioeconomic value. In deprived communities of the Eastern Amazon, forest restoration must be a process that combines environmental and social gains.

## Competing interests

The authors declare that they have no competing interests.

## Authors’ contributions

DC wrote the manuscript. DC, GXR, VLE and EGM conceived the study, and participated in its design and coordination. DC, CLF, EMO and GXR helped to draft the manuscript, collect the data and statistical analysis. All authors read and approved the final manuscript.
